# The role of EC-IC bypass in ICA blood blister aneurysms—a systematic review

**DOI:** 10.1007/s10143-020-01302-6

**Published:** 2020-04-21

**Authors:** Torstein R. Meling, Gildas Patet

**Affiliations:** 1 Department of Clinical Neurosciences, Division of Neurosurgery, Geneva University Hospitals, Geneva, Switzerland; 2 Faculty of Medicine, University of Geneva, Geneva, Switzerland; 3 Service de Neurochirurgie, Hôpitaux Universitaires de Genève, Rue Gabriel-Perret-Gentil 5, 1205 Genève, Switzerland; 4 Faculty of Medicine, University of Oslo, Oslo, Norway

**Keywords:** Blood blister–like aneurysm, Extracranial-to-intracranial bypass, Systematic review, Internal carotid artery, Neurosurgery

## Abstract

To perform a systematic review of extracranial-to-intracranial (EC-IC) bypass surgery with parent vessel trapping for blood blister–like aneurysms (BBAs) of the internal carotid artery (ICA) according to PRISMA guidelines. Search of PubMed using “bypass” [all fields] and “ICA” [all fields] or “internal carotid artery” [all fields] and (“blood blister–like aneurysm” [MeSH terms]. Thirty-four original articles were identified, of which 21 were excluded (treatment not including bypass or insufficient details on complications or clinical outcomes). Thirteen articles published between 2008 and 2019 were included, totaling 98 patients, with a median of 7.5 patients per article (range 1–17). Mean age was 53.3 years (range 23–80). The main techniques were external carotid artery to middle cerebral artery (ECA-MCA) in 81% and superficial temporal artery to MCA (STA-MCA) in 19%. The most common grafts were radial artery (74%) and STA (19%). The risk of intraoperative rupture varied from 0 to 75%, with a mean of 12%. With respect to clinical outcomes, the modified Rankin Scale (mRS) was not stated in 30% of the cases. When stated, mRS was ≤ 2 in 79%, mRS was 3–5 in 10%, and 4% had mRS 6 (death). We identified only 13 articles, with no prospective studies. Outcomes were better than generally reported for ruptured aneurysms, both with respect to poor outcome (mRS > 2) and in-hospital mortality, perhaps reflecting a selection bias. In general, the data reporting quality was low, precluding any firm conclusions, but EC-IC bypass with ICA trapping may be a valid treatment option for ruptured ICA BBAs.

## Introduction

Despite being rare, representing only 0.9–6.5% of all internal carotid artery (ICA) aneurysms, 1% of all intracranial aneurysms, and 0.5–2% of all ruptured intracranial aneurysms [1–3], blood blister–like aneurysms (BBAs) are challenging by nature of their fragility, small size, and clinical presentation. BBAs can occur in any intracranial artery but are more frequent in the supraclinoid ICA and then typically arise from the anterior wall [1, 3]. In contrast to saccular or berry-type aneurysms, BBAs are thought to emerge as a result of hemodynamic stress, atherosclerosis, or dissection [4–6], and their walls are composed of a thin layer of adventitia covering a focal defect in the intima and media and are located at non-branching sites of the cerebral circulation [3, 5, 7, 8].

Numerous treatments have been reported to overcome these specific and unique characteristics of BBAs [9]. Endovascular techniques include balloon-assisted coiling [10], stent-assisted coiling [11], covered stenting [11, 12], overlapping stents [13–15], flow diverters [16, 17], and parent vessel occlusion [3, 9]. There are different microsurgical options such as direct clipping [3, 9, 18, 19], clip wrapping [20, 21], trapping with or without an extracranial-to-intracranial bypass [19, 22–31], and primary suturing [19, 32, 33]. These techniques all carry various advantages and drawbacks [9]. Whereas the necessity of dual antiplatelet therapy may preclude endovascular therapy, clip reconstruction may fail either due to intraoperative aneurysm rupture, parent vessel stenosis, or focal vessel avulsion [3, 34].

Based on a cadaveric study, Ishikawa et al. [35] found that many BBAs are focal wall defects covered with thin fibrous tissue and that they are not true aneurysms. It was also found that many of them had an arterial dissection as their pathogenesis, making trapping of the ICA segment with the BBA the most effective surgical option [30, 36]. However, in the acute phase, ICA trapping leads to poor outcomes with a high risk of cerebral ischemia due to vasospasm and insufficient blood flow to the contralateral side despite a positive BTO [3].

Their friable nature means that BBAs are at risk of intraoperative rupture when direct clipping is attempted and intraoperative bleeding is frequently fatal [3, 37, 38]. In 2008, Meling et al. [3] demonstrated that ICA sacrifice in the acute stage of SAH inadvertently results in poor outcomes in patients not treated with combined revascularization surgery, and they hypothesized that high-flow extracrania- to-intracranial (EC-IC) bypass, when performed within the time-frame prior to the onset of vasospasms, may prevent vasospasm-induced cerebral infarcts and subsequent deaths associated with acute ICA sacrifice in SAH. Furthermore, an EC-IC bypass may lower the risk of intraoperative rupture since there is no dissection of the BBA [39]. Later, Kazumata et al. [27] demonstrated the proof of concept of EC-IC bypass in 20 patients with ICA BBAs by using RA graft bypass with parent vessel sacrifice during the acute phase of SAH. Since then, the use of an EC-IC bypass from the external carotid artery (ECA) or the superficial temporal artery (STA) to the middle cerebral artery (MCA) has emerged as valuable adjuncts. The technique particularly used for flow replacement when ICA sacrifice occurs [28, 40] or as a protective bypass prior to exploration and intended clipping of the ICA BBA [27].

The aim of this systematic review was to analyze the advantages and drawbacks IC-EC bypass in the treatment of ICA BBAs.

## Material and methods

**Fig. 1:**
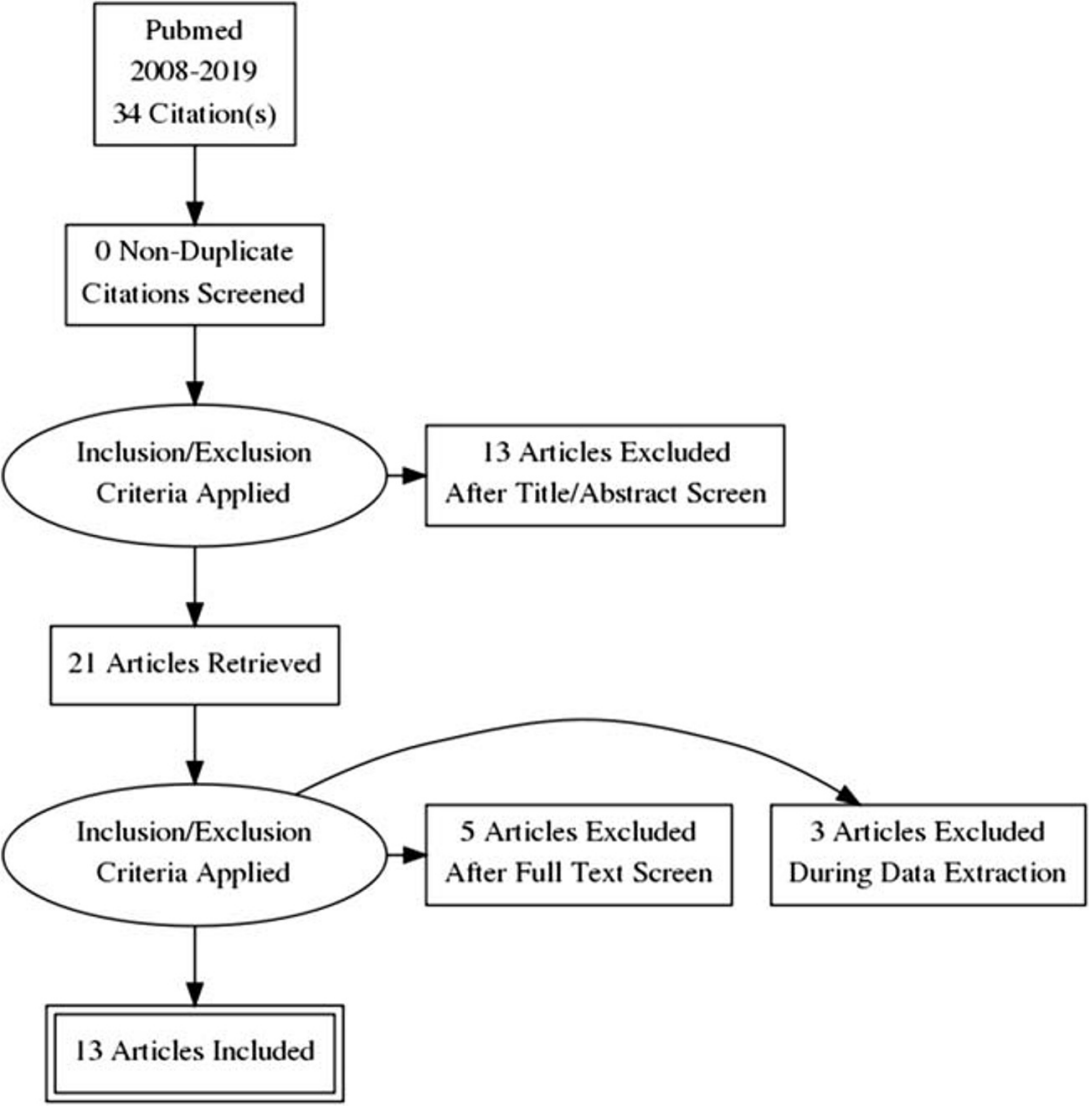
PRISMA flow diagram

A search of PubMed databases for the last 11 years (from 2008 to 2019) using the following criteria, “bypass” [all fields] and “ICA” [all fields] or “internal carotid artery” [all fields] and (“blood blister–like aneurysm” [MeSH terms], was performed. Basic inclusion filters were English language and articles providing information on the type of bypass used, complications, and clinical outcomes. Articles not related to the use of bypass for the treatment of ICA BBA were excluded. A PRISMA flow diagram was created in order to analyze the recent literature (Fig. 1) [41].

## Results

The literature search identified 34 articles, 21 of which were excluded due to treatment not including a bypass, not being in English, or not giving detailed information about the complications or the clinical outcomes (Fig. 1). The final 13 articles were compared with respect to number of patients, patient age, gender, BBA localization, rupture status, preoperative clinical scores, intention of the bypass, type of bypass used, intraoperative rupture rates, complications, and postoperative clinical outcomes (Tables 1 and 2).

**Table 1:** Patient and aneurysm characteristics

Study	Number of pts	Age (years)	Gender	Localization	Side	Rupture status	Preop Fisher grade	Preop clinical grade
Strickland et al. [39]	4	Not stated	Not stated	Not stated	Right 1 Not stated 3	Ruptured 4	Grade 2: 1 Grade 3: 2 Grade 4: 1	HH 2: 2 HH 3: 1 HH 4: 1
Kazumata et al. [27]	14	Mean 51.2 Median 52.5 Range 29–73	Female 11 Male 3	Circumferential 1 Anterior 1 Medial 11 Posterior 1	Not stated	Ruptured 12 Not stated 2	Grade 2: 4 Grade 3: 10	WFNS 1: 3 WFNS 2: 7 WFNS 3: 1 WFNS 4: 3
Owen et al. [18]	3	Mean 40.7 Median 42 Range 36–44	Female 1 Male 2	Not stated	Not stated	Ruptured 3	Grade 3: 2 Grade 4: 1	HH 2: 1 HH 3: 1 HH 5: 1
Kamijo et al. [40]	5	Mean 64.2 Median 70 Range 51–77	Female 5	Not stated	Right 1 Left 4	Ruptured 5	Grade 3: 5	WFNS 2: 2 WFNS 3: 1 WFNS 4: 2
Yu-Tse et al. [42]	3	Mean 52.7 Median 52 Range 52–54	Female 2 Male 1	Anteromedial 3	Right 3	Ruptured 3	Grade 3: 3	WFNS 3: 3
Ishikawa et al. [35]	4	Mean 53.3 Median 58 Range 33–64	Female 4	Not stated	Left 1 Not stated 3	Ruptured 1 Not stated 3	Not stated	WFNS 1: 2 WFNS 2: 2
Baskaya et al. [23]	4	Mean 40.0 Median 40 Range 23–57	Female 1 Male 3	Anterior 1 Anteromedial 1 Posterior 1 Not stated 1	Left 1 Not stated 3	Ruptured 3 Not stated 1	Grade 3: 4	HH 4: 4
Balik et al. [34]	9	Mean 57.2 Median 54 Range 43–71	Female 5 Male 4	Anterior 6 Not stated 3	Right 5 Left 4	Ruptured 9	Grade 3: 9	WFNS 1: 1 WFNS 2: 1 WFNS 4: 1 WFNS 5: 6
Sorimachi et al. [43]	15	Mean 50.0 Median 47 Range 32–80	Female 5 Male 10	Anterior 2 Posterior 13	Right 10 Left 5	Ruptured 15	Grade 3: 15	WFNS 1: 10 WFNS 2: 2 WFNS 4: 2 WFNS 5: 1
Kikkawa et al. [28]	16	Mean 58.9 Median 62.5 Range 40–77	Female 9 Male 7	Not stated	Not stated	Not stated	Not stated	WFNS 1: 2 WFNS 2: 5 WFNS 3: 2 WFNS 4: 4 WFNS 5: 3
Yang et al. [44]	3	Mean 55.0 Median 53 Range 53–59	Female 3	Anterior 3	Not stated	Ruptured 3	Not stated	HH 2: 2 HH 4: 1
Kawashima et al. [26]	1	Mean 32 Median 32	Female 1	Lateral 1	Right	Ruptured 1	Not stated	HH 2: 1
Aihara et al. [22]	17	Mean 52.4 Median 5 Range 28–67	Female 11 Male 6	Anterior 8 Lateral 9	Not stated	Not stated	Not stated	HH 1: 3 HH 2: 7 HH 3: 3 HH 4: HH 5: 2

**Table 2:** Bypass characteristics, complications, and clinical outcomes

Study	Type of bypass	Type of donor graft	Intention of bypass	Patency of bypass	Intraop. rupture	Complications	Glasgow Outcome Scale	Modified Rankin Scale
Strickland et al. [39]	ECA-M2: *n* = 3 STA-M2: *n* = 1	RA: *n* = 3 STA: *n* = 1	First intention before clipping/ligation: *n* = 4	Not stated	*n* = 0/4	Cerebral infarction: *n* = 1 Hydrocephalus/VP-shunt: *n* = 2	Good recovery: *n* = 3 Severe disability: *n* = 1	Not stated
Kazumata et al. [27]	ECA-M2: *n* = 12 STA-M2: *n* = 2	RA: 12 STA: *n* = 2	Not stated	Good: *n* = 14	*n* = 0/14	Cerebral infarction: *n* = 6 Hydrocephalus/VP-shunt: *n* = 6	Good recovery: *n* = 12 Severe disability: *n* = 2	mRS 0: *n* = 6 mRS 1: *n* = 3 mRS 2: *n* = 3 mRS 4: *n* = 2
Owen et al. [18]	ECA-M2: *n* = 1 STA-M2: *n* = 2	RA: *n* = 1 STA: *n* = 2	First intention before clipping/ligation: 1 Rescue for intra-op BBA rupture: *n* = 2	Not stated	*n* = 2/3	Cerebral infarction: *n* = 2 Death: *n* = 1	Good recovery: *n* = 1 Severe disability: *n* = 1 Death: *n* = 1	mRS 2: *n* = 1 mRS 3: *n* = 1 mRS 6: *n* = 1
Kamijo et al. [40]	ECA-M2: *n* = 5	RA: *n* = 5	First intention before clipping/ligation: *n* = 5	Good: *n* = 5	*n* = 0/5	Vasospasm: *n* = 4	Good recovery: *n* = 5	Not stated
Yu-Tse et al. [42]	ECA-M2: *n* = 3	RA: *n* = 3	First intention before clipping: *n* = 3	Not stated: *n* = 2 Poor: *n* = 1	*n* = 0/3	Hydrocephalus/VP-shunt: *n* = 2	Good recovery: *n* = 1 Severe disability: *n* = 2	mRS 1: *n* = 1 mRS 4: *n* = 1 mRS 5: *n* = 1
Ishikawa et al. [35]	ECA-M2: *n* = 4	RA: *n* = 4	First intention before clipping/ligation: *n* = 4	Good: *n* = 4	*n* = 3/4	Hydrocephalus/VP-shunt: *n* = 1	Good recovery: *n* = 4	mRS 0: *n* = 4
Baskaya et al. [23]	ECA-M2: *n* = 4	RA: *n* = 4	First intention before clipping/ligation: *n* = 4	Not stated: *n* = 3 Good: *n* = 1	*n* = 1/4	Vasospasm: *n* = 3 Death: *n* = 1	Good recovery: *n* = 3 Death: *n* = 1	mRS 1: *n* = 3 mRS 6: *n* = 1
Balik et al. [34]	STA-M2: *n* = 9	STA: *n* = 9	First intention before clipping/ligation: *n* = 9	Good: *n* = 9	*n* = 2/9	Contusion/meningitis: *n* = 1 Cerebral infarction: *n* = 2	Good recovery: *n* = 7 Severe disability: *n* = 2	mRS 0: *n* = 1 mRS 1: *n* = 4 mRS 2: *n* = 2 mRS 4: *n* = 1 mRS 5: *n* = 1
Sorimachi et al. [43]	ECA-M2: *n* = 14 STA-M2: *n* = 1	RA: *n* = 13 SVG: *n* = X1 STA: *n* = 1	First intention before clipping: *n* = 15	Good: *n* = 15	Not stated	Hydrocephalus/VP-shunt: *n* = 4 Vasospasm: *n* = 3	Good recovery: *n* = 13 Severe disability: *n* = 2	mRS 0: *n* = 11 mRS 1: *n* = 1 mRS 2: *n* = 1 mRS 4: *n* = 1 mRS 5: *n* = 1
Kikkawa et al. [28]	ECA-M2: *n* = 16	RA: *n* = 16	First intention before clipping/ligation: *n* = 16	Good: *n* = 16	*n* = 0/16	Severe vasospasm: *n* = 2 Cererabal infarction: *n* = 3 Hydrocephalus/VP-shunt: *n* = 1	Good recovery: *n* = 10 Mild disability: *n* = 4 Severe disability: *n* = 2	Not stated
Yang et al. [44]	ECA-M2: *n* = 3	RA: *n* = 2 SVG: *n* = 1	First intention before clipping [3]	Good: *n* = 3	*n* = 0/3	Motor aphasia: *n* = 1	Good recovery: *n* = 2 Severe disability: *n* = 1	Not stated
Kawashima et al. [26]	ECA-M2: *n* = 1	SVG: *n* = 1	BBA recurrence after clipping: *n* = 1	Good: *n* = 1	*n* = 0/1	Cerebral infarction: n = 1	Good recovery: *n* = 1	Not stated
Aihara et al. [22]	ECA-M2: *n* = 13 STA-M2: *n* = 4	RA: *n* = 11 SVG: *n* = 2 STA: *n* = 4	First intention before clipping/ligation: *n* = 17	Good: *n* = 17	*n* = 2/17	Severe vasospasm: n = 4 Cerebral infarction: n = 4 Death: n = 2	Good recovery: *n* = 9 Mild disability: *n* = 6 Death: *n* = 2	mRS 0: *n* = 7 mRS 1: *n* = 2 mRS 2: *n* = 5 mRS 4: *n* = 1 mRS 6: *n* = 2

In summary, the analysis showed that (Table 3):

1. The total number of patients was 98, with a median of 7.5 patients per article included in this review (range 1–17).
2. The patients’ age was 53.3 years (range 23–80).
3. There was a predominance of females with 62% vs. 38% males.
4. The anterior, medial, or anteromedial wall of ICA was the most common location of the BBA with 56% compared with 24% of the posterior wall and 16% on the lateral wall. The localization was not stated in 37% of the cases (Table 3).
5. With respect to laterality, this information was not available in 63% of the cases (Table 3). The BBAs were located on the right side in 58% when stated.
6. All the cases were ruptured when explicitly stated, but information was lacking for 39 patients (40%) (Table 3). Likewise, the preoperative Fisher grade was not stated in 42%.
7. With respect to the preoperative clinical condition, a Hunt and Hess score or a WFNS score was not stated in 67 and 33% of the cases, respectively. When reported, the clinical scores varied widely between individuals, from pauci- or asymptomatic (WFNS score < 3) in 56% to poor neurological condition (WFNS score ≥ 3) to) in 44% (Table 3).
8. The intention of the bypass was *pre hoc* in 84%, post hoc in 2%, and not stated in 14%. The intention of *pre hoc* bypasses were either protective (followed by aneurysm clipping) or replacement (followed by ICA trapping) (Table 2).
9. The main techniques used for IC-EC bypass were ECA-MCA (81%) and STA-MCA (19%). The most common grafts were radial artery (RA) (74%) and superficial temporal artery (STA) (19%), while the saphenous vein (SA) was less commonly used (7%) (Table 3).
10. The risk of intraoperative rupture varied according to the different series. Indeed, some authors had a 75% intraoperative rupture rate, while others reported none. Overall, the mean intraoperative rupture was 12%.
11. In terms of complications, 10% of the patients developed vasospasms, and 23% had infarcts or contusions (Table 3).
12. With respect to clinical outcomes, the modified Rankin Scale (mRS) was not stated in 30% of the cases. When stated, the outcome was good (mRS score ≤ 2) in 79%, whereas 10% had moderate to severe disabilities (mRS 3–5) and 4% had mRS 6 (death). When using the Glasgow Outcome Scale, good recovery was seen in 71%, mild disability in 10%, severe disability in 13%, and death in 4% (Table 3).

**Table 3:** Summary of results

Number of patients	Total: 98
	Range: 1–17
	Mean: 7.5 per study
Age	Not stated: 4/98 (4.1%)
	Mean: 53.3 years
	Median: 53 years
	Range: 23–80 years
Gender	Not stated: 4/98 (4%)
	Female: 58/94 (62%)
	Male: 36/94 (38%)
Localization	Not stated: 36/98 (37%)
	Circumferential: 1/63 (2%)
	Anterior: 21/63 (33%)
	Anteromedial: 4/63 (6%)
	Medial: 11/63 (17%)
	Lateral: 10/63 (16%)
	Posterior: 15/63 (24%)
Side	Not stated: 62/98 (63%)
	Right: 21/36 (58%)
	Left: 15/36 (42%)
Rupture status	Not stated: 39/98 (40%)
	Ruptured: 59/59 (100%)
Preop Fischer grade	Not stated: 41/98 (42%)
	Grade 1: 0/57 (0%)
	Grade 2: 5/57 (9%)
	Grade 3: 50/57 (88%)
	Grade 4: 2/57 (4%)
Preop Hunt and Hess score	Not stated: 66/98 (67%)
	Grade 1: 3/32 (9%)
	Grade 2: 13/32 (41%)
	Grade 3: 5/32 (16%)
	Grade 4: 8/32 (25%)
	Grade 5: 3/32 (9%)
Preop WFNS grade	Not stated: 32/98 (33%)
	Grade 1: 18/66 (15%)
	Grade 2: 19/66 (29%)
	Grade 3: 7/66 (11%)
	Grade 4: 12/66 (18%)
	Grade 5: 10/66 (15%)
Type of bypass	Not stated: 0/98 (0%)
	ECA-M2: 79/98 (81%)
	STA-M2: 19/98 (19%)
Type of donor graft	Not stated: 0/98 (0%)
	RA: 74/98 (76%)
	SV: 7/98 (7%)
	STA: 19/98 (19%)
Intention of bypass	Not stated: 14/98 (14%)
	First intention before clipping: 81/84 (96%)
	Rescue for intraop. BBA rupture 2/84 (2%)
	BBA recurrence after clipping 1/84 (1%)
Patency of bypass	Not stated: 12/98 (12%)
	Good: 85/86 (99%)
	Poor: 1/86 (1%)
Intraoperative rupture	Not stated 15/98 (15%)
	Yes 10/83 (12%)
	No 73/83 (88%)
Complications	Not stated: 0/98 (0%)
	Vasospasm: 10/98 (10%)
	Infarct/stroke: 21/98 (21%)
	Contusion/motor aphasia: 2/98 (2%)
	HC requiring VP shunt: 16/98 (16%)
	Deaths: 3/98 (3%)
Glasgow Outcome Scale	Not stated: 0/98 (0%)
	Good recovery: 71/98 (71%)
	Mild disability: 10/98 (10%)
	Severe disability: 13/98 (13%)
	Death: 4/98 (4%)
Modified Rankin Scale	Not stated: 29/98 (30%)
	mRS 0: 29/69 (42%)
	mRS 1: 14/69 (20%)
	mRS 2: 12/69 (17%)
	mRS 3: 1/69 (1%)
	mRS 4: 6/69 (9%)
	mRS 5: 3/69 (4%)
	mRS 6: 4/69 (6%)

## Discussion

This systematic review analyzed the use of IC-EC bypass in the treatment of ICA BBAs. We identified only 13 articles (Fig. 1, Tables 1 and 2), and in general, the quality of the data reporting was low (Table 3). There were no prospective studies identified. In the papers included, the total number of patients was 98, with a mean of 7.5 patients per article (range 1–17), reflecting the small population affected by ICA BBAs and inferring that statistical meta-analysis would be futile.

BBAs are more frequently seen at a younger age [3, 7, 20, 45] and occur more frequently in females [3, 20, 46, 47] and on the right ICA [3, 7, 20, 47, 48] than the typical saccular ICA aneurysms. In the papers reviewed, mean patient age was 53.3 years, and 62% of the patients were females (Table 1). The BBAs were located on the right side in 58% of cases, and the anterior or anteromedial wall of ICA was the most common location of the BBA with 56%. This is in line with previous reports [3, 7, 20].

The majority of the cases were ruptured, but in 40% of the cases, the rupture status was not stated (Table 1). This is in line with previous reports where the vast majority of BBAs present with SAH [3, 7, 20, 39, 49]. With respect to preoperative clinical condition, neither a Hunt and Hess score nor a WFNS score was stated in 67 and 33% of cases, respectively. When stated, the clinical presentation varied widely between individuals, from a- or pauci-symptomatic (WFNS score < 3) in 56% to poor neurological condition (WFNS score ≥ 3) to) in 44%, which is in line with the scientific literature on aneurysmal SAH in general [50].

With respect to the intention of EC-IC bypass, it can be used *pre hoc* or up-front when ICA trapping is planned or when a primary clip reconstruction was planned preoperatively, but the intraoperative inspection suggests an excessive friability of the BBA [18, 27, 28, 35, 43]. Despite requiring more time and entails increased complexity, this strategy reduces the risk of a devastating intraoperative hemorrhage [18, 35, 39]. In our review, the intention of the bypass was *pre hoc* in 84%, post hoc in 2%, and unknown in 14%. A *pre hoc* intention was either protective (followed by aneurysm clipping) or replacement (followed by ICA trapping).

The bypass graft types were RA (74%), STA (19%), and SV (7%). The choice of bypass graft might be based on surgeon preference or estimations of collateral flow, as assessed by a preoperative angiography [51]. However, although donor graft selection remains debated [52], a RA graft is often preferred [27, 40]. Arteries are valve-free and may remain open even at low flow rates, qualities that might contribute to superior long-term patency rates of RA grafts [44]. Furthermore, RA and STA grafts are less vulnerable to traumatic injury during harvesting, and arterial grafts are generally more resistant to kinking and torsion than venous grafts. However, a SV graft has the advantages of its long length, easy manipulability, and high-flow conduit [53], but the discrepancy in diameter between a SV donor graft and a MCA recipient vessel is a significant limitation as it may create turbulent flow at the anastomosis site and cause delayed graft occlusion [44]. In this review, all graft types had good patency rates, and there were no significant differences between them in terms of outcomes (Table 3). Ischemic stroke was seen in 21% of the patients (Table 3).

Severe symptomatic vasospasms may develop in SAH patients [34, 50], and if it occurs in the territory of a major cerebral artery, cerebral angioplasty, selective intra-arterial vasodilator therapy, or both can be considered [54]. The bypass conduit can potentially be used as a route of endovascular drug administration to treat vasospasm when it is necessary [35]. STA-MCA bypasses allow for the use of intra-arterial vasodilators, whereas RA grafts can even be used for super-selective intra-arterial drug instillations, and large conduits like the SV grafts may even allow for catheter angioplasty via the graft. Furthermore, considering the risk of vasospasm and the deleterious effects of such in the acute stage of a SAH [3], a high-flow ECA-MCA bypass with RA or SV donor grafts may be advantageous over a low-flow STA-MCA bypass in maintaining perfusion pressure and cerebral blood flow in the ICA after trapping [27, 40]. In this systematic review, high-flow bypass was used in 81% of the cases (Table 3).

In the papers reviewed, the risk of intraoperative aneurysm rupture varied between 0 and 75% (Table 2). Overall, the mean intraoperative rupture rate was 12% (Table 3), which is much lower than that associated with direct microsurgical clipping that typically carries a 30–50% risk of intraoperative rupture [3, 7, 18, 33, 46, 55–60] This indicates a significant risk-reducing effect of an EC-IC bypass in ruptured ICA BBAs [27, 39], and an intraoperative rupture rate of 12% is on par with the clip-wrap technique, albeit in small case series [19, 20, 48, 56, 61].

With respect to clinical outcomes, the data quality was generally very poor as the modified Rankin Scale (mRS) was not stated in 30% of the cases (Table 3). When stated, the outcome was good (mRS score ≤ 2) in 79%, whereas 10% had moderate to severe disabilities (mRS 3–5), and 4% had mRS 6 (death). When the Glasgow Outcome Scale was used, good recovery was seen in 71%, mild disability in 10%, severe disability in 13%, and death in 4% (Table 3). These results are better than generally reported for acute SAH, both with respect to poor outcome (mRS > 2) and the in-hospital mortality [62, 63], perhaps reflecting a selection bias.

Although EC-IC bypass with ICA trapping may be a valid treatment option for ruptured ICA BBAs, many clinical series have employed various surgical and endovascular techniques, reflecting a lack of solid evidence of superiority of any method [3, 9, 20, 42, 58]. Whereas microsurgery offers superior obliteration rates and neurological outcomes on par with endovascular treatments [9], it comes at the price of a higher complication rates. In contrast, endovascular therapy offers superior safety and provides functional outcomes comparable with surgery [9, 46, 57], albeit at a higher financial cost both in terms of retreatments, regular angiographic controls, and device costs. Multilayer flow-diverting stents appear to be a promising strategy, but at present, flow diversion devices (FDDs) lack sufficient evidence from trials and have only been used for a short period of time [9, 46], and the double antiplatelet therapy is still a major constraint in the setting of SAH.

## Conclusions

In this systematic review, we identified only 13 articles, with no prospective studies. Outcomes were better than generally reported for ruptured aneurysms, both with respect to poor outcome (mRS > 2) and in-hospital mortality [62, 63], perhaps reflecting a selection bias. Furthermore, in general, the data reporting quality was low, precluding any firm conclusions, but EC-IC bypass with ICA trapping may be a valid treatment option for ruptured ICA BBAs.
